# Dr. Claud Muirhead

**Published:** 1910-07-09

**Authors:** 


The death is announced at Edinburgh of Dr. Claud Muirhead, in his 75th year. He studied medicine at Berlin, Vienna, and Paris, took the M.D. at Edinburgh in 1862, and in 1865 became a Fellow of the R.C.P.Edin. His appointments included those of physician (afterwards consultant) to the Royal Infirmary, Edinburgh, to the Deaconess Hospital, Edinburgh, and physician (afterwards consultant) to the Edinburgh New Town Dispensary. He was also ex-President of the Royal Medical Society, Edinburgh. Besides occasional contributions to the medical papers, Dr. Muirhead wrote also "The Causes of Death Among the Assured, 1874-94."

